# Towards an Integrated Mycorrhizal Technology: Harnessing Mycorrhiza for Sustainable Intensification in Agriculture

**DOI:** 10.3389/fpls.2016.01625

**Published:** 2016-10-27

**Authors:** Matthias C. Rillig, Moisés A. Sosa-Hernández, Julien Roy, Carlos A. Aguilar-Trigueros, Kriszta Vályi, Anika Lehmann

**Affiliations:** ^1^Institut für Biologie, Plant Ecology, Freie Universität BerlinBerlin, Germany; ^2^Berlin-Brandenburg Institute of Advanced Biodiversity ResearchBerlin, Germany

**Keywords:** agricultural sustainability, mycorrhiza, technology, biodiversity, communication

## Background

### Sustainability in agriculture

In order to meet future needs of a growing human population and to achieve food security in the context of climate change, food production will likely need to increase—among other measures—while at the same time minimizing negative environmental impact (Foley et al., 2011). Sustainable intensification of agriculture (Garnett et al., 2013; Pretty and Bharucha, 2014; Andres and Bhullar, 2016; Gunton et al., 2016), sometimes also called ecological intensification, is likely to include key aspects of conservation agriculture (e.g., Hobbs et al., 2008; Giller et al., 2015). Pillars of conservation agriculture (FAO, 2015) are no-till practices (Pittelkow et al., 2015), continuous crop cover (by various means, for example cover crops) and diversification practices (multi-cropping and crop rotations; Ponisio et al., 2015).

### The potential role of mycorrhiza in sustainable agriculture

There is a steadily growing appreciation of the integral importance of soil life in agricultural sustainability (e.g., Mäder et al., 2002; Wagg et al., 2014; Bender et al., 2016), including plant-symbiotic associations. Among these symbioses a prominent player is mycorrhiza, the widespread symbiotic association of fungi with plant roots (Smith and Read, 2008). Much has been written about the role of mycorrhiza in agroecosystems, in particular about arbuscular mycorrhiza (AM), formed by fungi in the phylum Glomeromycota, to which this paper mostly refers. AM fungi are obligate symbionts (they need a living host during their entire life cycle) with tight regulation of carbon-for-nutrients exchange between the host and the fungus. During their evolution, AM fungi have lost the enzymatic ability to degrade carbon compounds (Tisserant et al., 2013), which prevents them from becoming necrotrophic pathogens (the most common type of root-fungal pathogens). The AM symbiosis has been much discussed in the context of agriculture, (i) because AM is the dominant mycorrhiza formed by most crops (an exception being for example crops in the Brassicaceae); (ii) because of the potentially positive, multifunctional role of AM in plant nutrition, pathogen protection, stress tolerance and soil structure provision (Hamel, 1996; Smith and Read, 2008; Gianinazzi et al., 2010; Leifheit et al., 2014); (iii) because many agricultural practices (e.g., tillage, fertilization, non-host crops) tend to negatively affect AM fungal abundance and diversity, thus potentially affecting functioning; and (iv) because AM fungi can be managed.

### Defining mycorrhizal technology

The focus in applied research on mycorrhiza in sustainable agriculture could be circumscribed as developing mycorrhizal technology. Clearly most easily recognized as a mycorrhizal technology is the production and application of mycorrhizal fungal inoculum (Gianinazzi et al., 2002; Vosatka et al., 2012; Solaiman et al., 2014), directly addressing the decline in mycorrhizal abundance in agricultural fields. Inoculation can have demonstrable yield benefits, powerfully documented by the recent, very in-depth study by Hijri (2016) analyzing 231 potato field trials. Nevertheless, we argue here that this should not be the exclusive focus of next-generation mycorrhizal technology. A recent analysis concluded that one of the most striking aspects of sustainable agricultural intensification is an “increase in knowledge per hectare” (Buckwell et al., 2014), i.e., a better understanding of how to achieve resource-efficient agroecosystems with minimal environmental impacts in any given location. This is a very useful conceptualization that helps frame what mycorrhizal technology could or should increasingly mean.

We here propose a definition of “mycorrhizal technology” as the set of measures to optimize local mycorrhizal abundance and diversity in terms of functioning for attaining sustainability of agroecosystems. Optimization here means increasing mycorrhizal benefits (in terms of yield and sustainability of other ecosystems processes) within given socioeconomic constraints, i.e., there will always be practical limits at the farm level to achieving the full theoretical potential (e.g., Lamarque et al., 2014). This definition includes sustainability as a clear goal and it is inclusive of many approaches discussed in the following.

## Mycorrhizal technology: components and research needs

### Components of an inclusive mycorrhizal technology

**Figure 1:**
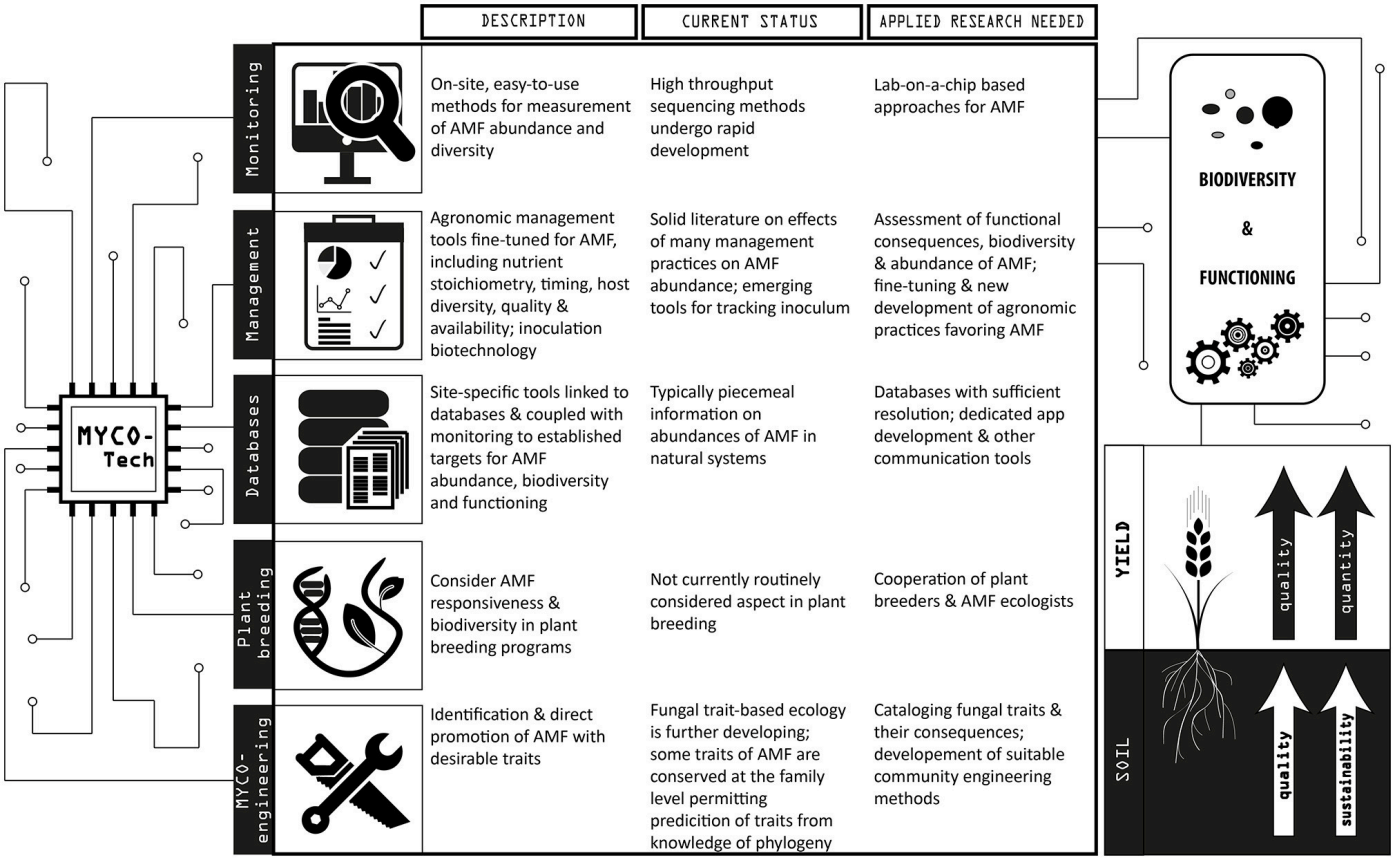
**Depiction of components of mycorrhizal technology, their current status and specific research needs**. Development of these various components of mycorrhizal technology has the goal to enhance abundance and diversity of arbuscular mycorrhizal (AM) fungi, leading to enhanced functioning. Functioning of mycorrhiza is defined in the context of crop performance (yield and quality), as well as agroecosystem sustainability (here symbolized by soil quality and sustainability).

We propose some key elements of an inclusive mycorrhizal technology (Figure 1): monitoring, agricultural management, database tools, plant breeding and ecological engineering of communities of mycorrhizal fungi (“myco-engineering”) and their associated microbiota. *Monitoring* refers to assessment of the abundance (in roots and soil) and diversity of mycorrhizal fungal abundance in the field. *Management* represents a complex set of tools that can impact abundance and diversity of mycorrhiza, including agronomic practices with known effects on mycorrhiza (e.g., crop rotations, tillage, fertilizer and other additions), and also more directly targeted approaches, such as inoculation with fungal strains or mycorrhization helper-bacteria. *Databases* serve as dynamic repositories of site-specific information linking site parameters with mycorrhizal abundance and functioning. *Plant breeding* is an important component, since mycorrhizal fungi require suitable hosts. And *myco-engineering* denotes an approach grounded in community ecology with the goal to promote members of the mycorrhizal fungal community with desirable traits. Inroads toward the development of these various components of a mycorrhizal technology already have been made, but there are still varying demands for research and development (Figure 1).

The real power of this set of measures may become most apparent when several components can interact. Agricultural management practices, for example, offer a rich opportunity for interventions affecting mycorrhizal fungi. The AM symbiosis responds sensitively to nutrient ratios (Johnson, 2010), and since AM fungal phylotypes associated with certain N:P ratios may be preferentially lost upon cultivation (Verbruggen et al., 2015), tightly managing nutrient stoichiometry in agricultural systems, including the amount, timing and element ratios of nutrient additions will be an important priority—of course always with a view toward ensuring yield. Easy monitoring of the abundance (and functioning) of AM fungi, ideally in real time, possibly the biggest bottleneck at the moment, would allow detection of deleterious management practices, permitting precise responses at the farm level. These data, when combined over many fields and over time, can feed databases to iteratively fine-tune local management interventions. Choice of crop genotypes, including cover crops, will directly determine host quality for the symbiosis, and thus fungal abundance; and here collaboration of mycorrhizal researchers with plant breeders offers an immense opportunity to have available genotypes that support mycorrhiza. Already, plant breeders are increasingly looking to the roots (Bishopp and Lynch, 2015).

Microbial community engineering (sensu Mueller and Sachs, 2015) is a rapidly developing field in microbial ecology. This technique relies on the selection over multiple microbial generations of entire communities that outperform in a particular function. It has already been used in the selection of bacterial communities, thus similar techniques can be brought to bear on communities of AM fungi, or, perhaps even more importantly, AM fungi and the microbes associated with their extraradical mycelium. Engineering entire consortia, with AM fungi as a part of this larger system, may be very promising, since it is clear that AM functionality may depend on interactions with many other soil organisms (and *vice versa*). For example, in terms of delivery of nutrients to the crop plant, AM fungi can closely interact with and profit from a consortium of bacteria and protozoa (Koller et al., 2013), and in terms of their role in soil aggregation, AM fungi can have effects complementary to those of other soil biota, for example collembola (Siddiky et al., 2012). In fact, the entire body of literature on mycorrhiza helper bacteria (e.g., Frey-Klett et al., 2007) also serves to illustrate the complex interdependencies between AM fungal functioning and the soil microbiome.

Hopefully, more tools can be added to this list as our knowledge of mycorrhizal ecology and evolution grows.

Given the importance of “knowledge per hectare,” a crucial component of mycorrhizal technology is two-way communication with stakeholders by various means including workshops, manuals and data-driven apps. Applications could be developed through several stages of technological advancement. The simplest apps would provide the user (farmer) with field-specific information and suggestions based on user input, relying on evidence based information. Social applications could connect farmers, mycorrhizal consultants and customers. More advanced apps would include live monitoring of field variables and mycorrhizae through sensors and, in the future, real time sequence analysis or abundance analysis.

### Research needs

Development of new mycorrhizal technologies needs to be supported by research, and specific research needs for the various components of technology are detailed in Figure 1. In addition to these, there are some general research needs that are crucial, which we discuss here.

First, we need a better understanding of the *relative contribution* of mycorrhiza to any aspect of sustainability. That is, achieving a comprehensive view of all major hypothesized causal pathways by which mycorrhiza influence sustainability, including their interactions with other soil biota. For example, this means measuring also other organism groups, especially if those are also typically important in a given process, for example for nutrient cycling (symbiotic and non-symbiotic diazotrophs, phosphate mobilizers, nitrifiers, ammonia oxidizers) or for soil aggregation (soil animals, roots, bacteria, saprobic fungi). The same also applies to assessing other causal influences (site factors) in order to assess mycorrhizal contributions to yield increase, soil aggregation, crop quality or other parameters. The use of statistical tools such as variance partitioning or structural equation modeling is required to attribute relative effect sizes of mycorrhiza given all the other causal pathways.

A second research priority relates to defining which parameters influence mycorrhizal effectiveness. This will help prevent other agricultural management approaches from interfering with the mycorrhizal-mediated benefit. Data demonstrating that mycorrhizas are not as important as previously believed in some circumstances (e.g., Ryan and Kierkegaard, 2012) or for some processes are ultimately very useful in providing realistic expectations for stakeholders and for setting management priorities.

Third, there is a need to expand the response variables for documenting mycorrhizal effects. For example, plenty of data exist for direct plant growth responses, but dramatically less knowledge exists for nutrient and micronutrient contents in the relevant (consumed) tissues (e.g., Lehmann et al., 2014; Lehmann and Rillig, 2015). When considering multiple mycorrhizal-influenced parameters it may also become evident that there are conflicts, i.e., mycorrhiza may positively influence some plant traits and others negatively (e.g., pollination and nutrient uptake; Barber et al., 2013). Carefully documenting such tradeoffs and understanding their mechanistic basis would be crucial and could advance mycorrhizal technology.

## What is the way forward?

Applied research and development (*sensu* Courchamp et al., 2015) is needed to support mycorrhizal technology, which needs to be funded from a variety of sources. The private sector, i.e., companies, are currently investing primarily development for the production and delivery of mycorrhizal inoculum. We think this needs to broaden with the aim of developing knowledge- and evidence-based mycorrhizal technologies. Based on ever-increasing amounts of information, development of and investment in ideally open-access information technology will be crucial.

Currently, funding the development of mycorrhizal technology suffers is hampered by unrealistic expectations, and on the other hand, a perception of frequent failures related to context-dependence of mycorrhizal effects. Both prevent widespread appreciation of the role of mycorrhiza in production agriculture. Here, there is an urgent need for effective communication aimed at clarifying the important distinction between uncertainty and variability (Lehmann and Rillig, 2014): they are not the same, but often are perceived, by both scientists and stakeholders, as being synonymous. Mycorrhizal effects may be highly context-dependent (Hoeksema et al., 2010), i.e., variable, but a lot of this variability can already be understood (and may ultimately be much more accurately captured in site-specific information delivery and apps) and is thus not uncertainty, i.e., not unpredictable. Mycorrhizal technology can help improve the proportion of variability that can be explained in a site-specific manner.

## Conclusions

Mycorrhizal fungi occur as communities of organisms, embedded in complex biotic interactions in the soil, responding dynamically to an array of environmental cues and management factors - this is a complex ecological and also evolutionary (Verbruggen and Kiers, 2010) situation that lacks a “one size fits all” solution. We need to develop the appropriate technology, with the components as defined here, and with a view toward social sustainability, matching this complexity to better harness the mycorrhizal symbiosis for a sustainable functioning of agroecosystems.

## Author contributions

MR: Wrote the first draft of the paper; MS, JR, CA, KV, and AL: Contributed ideas and text, and AL designed the figure.

### Conflict of interest statement

The authors declare that the research was conducted in the absence of any commercial or financial relationships that could be construed as a potential conflict of interest.

## References

[B1] Andres C. Bhullar G. S. (2016). Sustainable intensification of tropical agro-ecosystems: need and potentials. Front. Environ. Sci. 4:5 10.3389/fenvs.2016.00005

[B2] Barber N. A. Kiers E. T. Theis N. Hazzard R. V. Adler L. S. (2013). Linking agricultural practices, mycorrhizal fungi, and traits mediating plant-insect interactions. Ecol. Appl. 23, 1519–1530. 10.1890/13-0156.1 24261037

[B3] Bender S. F. Wagg C. van der Heijden M. G. A. (2016). An underground revolution: biodiversity and soil ecological engineering for agricultural sustainability. Trends Ecol. Evol. 31, 440–452. 10.1016/j.tree.2016.02.016 26993667

[B4] Bishopp A. Lynch J. P. (2015). The hidden half of crop yields. Nat. Plants 1:15117. 10.1038/nplants.2015.117 27250548

[B5] Buckwell A. Nordang Uhre A. Williams A. Polakova J. Blum W. E. H. Schiefer J. (2014). Sustainable Intensification of European Agriculture. Brussels: RISE.

[B6] Courchamp F. Dunne J. A. Le Maho Y. May R. M. Thébaud C. Hochberg M. E. (2015). Fundamental ecology is fundamental. Trends Ecol. Evol. 30, 9–16. 10.1016/j.tree.2014.11.005 25481619

[B7] FAO, (2015). Conservation Agriculture. Available online at: http://www.fao.org/ag/ca/index.html (Accessed November 11, 2015).

[B8] Foley J. A. Ramankutty N. Brauman K. A. Cassidy E. S. Gerber J. S. Johnston M.. (2011). Solutions for a cultivated planet. Nature 478, 337–342. 10.1038/nature10452 21993620

[B9] Frey-Klett P. Garbaye J. Tarkka M. (2007). The mycorrhiza helper bacteria revisited. New Phytol. 176, 22–36. 10.1111/j.1469-8137.2007.02191.x 17803639

[B10] Garnett T. Appleby M. C. Balmford A. Bateman I. J. Benton T. G. Bloomer P.. (2013). Sustainable intensification in agriculture: premises and policies. Science 341, 33–34. 10.1126/science.1234485 23828927

[B11] Gianinazzi S. Gollotte A. Binet M. N. van Tuinen D. Redecker D. Wipf D. (2010). Agroecology: the key role of arbuscular mycorrhizas in ecosystem services. Mycorrhiza 20, 519–530. 10.1007/s00572-010-0333-3 20697748

[B12] Gianinazzi S. Schüepp H. Barea J. M. Haselwandter K. (2002). Mycorrhizal Technology in Agriculture - from Genes to Bioproducts. Basel: Birkhäuser Verlag.

[B13] Giller K. E. Andersson J. A. Corbeels M. Kirkegaard J. Mortensen D. Erenstein O.. (2015). Beyond conservation agriculture. Front. Plant Sci. 6:870. 10.3389/fpls.2015.00870 26579139 PMC4623198

[B14] Gunton R. M. Firbank L. G. Inman A. Winter D. M. (2016). How scalable is sustainable intensification? Nat. Plants 2:16065. 10.1038/nplants.2016.65 27243658

[B15] Hamel C. (1996). Prospects and problems pertaining to the management of arbuscular mycorrhizae in agriculture. Agricu. Ecosyst. Environ. 60, 197–210. 10.1016/S0167-8809(96)01071-7

[B16] Hijri M. (2016). Analysis of a large dataset of mycorrhiza inoculation field trials on potato shows highly significant increases in yield. Mycorrhiza 26, 209–214. 10.1007/s00572-015-0661-4 26403242

[B17] Hobbs P. R. Sayre K. Gupta R. (2008). The role of conservation agriculture in sustainable agriculture. Philos. T. R. Soc. B. 363, 543–555. 10.1098/rstb.2007.2169 17720669 PMC2610169

[B18] Hoeksema J. D. Chaudhary V. B. Gehring C. A. Johnson N. C. Karst J. Koide R. T.. (2010). A meta-analysis of context-dependency in plant response to inoculation with mycorrhizal fungi. Ecol. Lett. 13, 394–407. 10.1111/j.1461-0248.2009.01430.x 20100237

[B19] Johnson N. C. (2010). Resource stoichiometry elucidates the structure and function of arbuscular mycorrhizas across scales. New Phytol. 185, 631–647. 10.1111/j.1469-8137.2009.03110.x 19968797

[B20] Koller R. Rodriguez A. Robin C. Scheu S. Bonkowski M. (2013). Protozoa enhance foraging efficiency of arbuscular mycorrhizal fungi for mineral nitrogen from organic matter in soil to the benefit of host plants. New Phytol. 199, 203–211. 10.1111/nph.12249 23534902

[B21] Lamarque P. Meyfroidt P. Nettier B. Lavorel S. (2014). How ecosystem services knowledge and values influence farmers' decision-making. PLoS ONE 9:e107572. 10.1371/journal.pone.0107572 25268490 PMC4182349

[B22] Lehmann A. Rillig M. C. (2015). Arbuscular mycorrhizal contribution to copper, manganese and iron nutrient concentrations in crops - A meta-analysis. Soil Biol. Biochem. 81, 147–158. 10.1016/j.soilbio.2014.11.013

[B23] Lehmann A. Veresoglou S. D. Leifheit E. F. Rillig M. C. (2014). Arbuscular mycorrhizal influence on zinc nutrition in crop plants - A meta-analysis. Soil Biol. Biochem. 69, 123–131. 10.1016/j.soilbio.2013.11.001

[B24] Lehmann J. Rillig M. (2014). Distinguishing variability from uncertainty. Nat. Clim. Change 4, 153–153. 10.1038/nclimate2133

[B25] Leifheit E. F. Veresoglou S. D. Lehmann A. Morris E. K. Rillig M. C. (2014). Multiple factors influence the role of arbuscular mycorrhizal fungi in soil aggregation – a meta-analysis. Plant Soil 374, 523–537. 10.1007/s11104-013-1899-2

[B26] Mäder P. Fliessbach A. Dubois D. Gunst L. Fried P. Niggli U. (2002). Soil fertility and biodiversity in organic farming. Science 296, 1694–1697. 10.1126/science.1071148 12040197

[B27] Mueller U. G. Sachs J. L. (2015). Engineering microbiomes to improve plant and animal health. Trends Microbiol. 23, 606–617. 10.1016/j.tim.2015.07.009 26422463

[B28] Pittelkow C. M. Liang X. Linquist B. A. van Groenigen K. J. Lee J. Lundy M. E.. (2015). Productivity limits and potentials of the principles of conservation agriculture. Nature 517, 365–370. 10.1038/nature13809 25337882

[B29] Ponisio L. C. M'Gonigle L. K. Mace K. C. Palomino J. de Valpine P. Kremen C. (2015). Diversification practices reduce organic to conventional yield gap. Proc. Roy. Soc. B 282:20141396. 10.1098/rspb.2014.1396 25621333 PMC4286047

[B30] Pretty J. Bharucha Z. P. (2014). Sustainable intensification in agricultural systems. Ann. Bot. 114, 1571–1596. 10.1093/aob/mcu205 25351192 PMC4649696

[B31] Ryan M. H. Kierkegaard J. A., (2012). The agronomic relevance of arbuscular mycorrhizas in the fertility of Australian extensive cropping systems. Agric. Ecosyst. Environ. 163, 37–53. 10.1016/j.agee.2012.03.011

[B32] Siddiky M. R. K. Schaller J. Caruso T. Rillig M. C. (2012). Arbuscular mycorrhizal fungi and collembola non-additively increase soil aggregation. Soil Biol. Biochem. 47, 93–99. 10.1016/j.soilbio.2011.12.022

[B33] Smith S. E. Read D. (2008). Mycorrhizal Symbiosis, 3rd Edn. San Diego, CA: Academic Press.

[B34] Solaiman Z. Abbott L. K. Varma A. (2014). Mycorrhizal Fungi: Use in Sustainable Agriculture and Land Restoration. Heidelberg: Springer.

[B35] Tisserant E. Malbreil M. Kuo A. Kohler A. Symeonidi A. Balestrini R.. (2013). Genome of an arbuscular mycorrhizal fungus provides insight into the oldest plant symbiosis. Proc. Natl. Acad. Sci.U.S.A. 110, 20117–20122. 10.1073/pnas.1313452110 24277808 PMC3864322

[B36] Verbruggen E. Kiers E. T. (2010). Evolutionary ecology of mycorrhizal functional diversity in agricultural systems. Evol. Appl. 3, 547–560. 10.1111/j.1752-4571.2010.00145.x 25567946 PMC3352509

[B37] Verbruggen E. Xiang D. Chen B. Xu T. Rillig M. C. (2015). Mycorrhizal fungi associated with high soil N:P ratios are more likely to be lost upon conversion from grasslands to arable agriculture. Soil Biol. Biochem. 86, 1–4. 10.1016/j.soilbio.2015.03.008

[B38] Vosatka M. Latr A. Gianinazzi S. Albrechtova J. (2012). Development of arbuscular mycorrhizal biotechnology and industry: current achievements and bottlenecks. Symbiosis 58, 29–37. 10.1007/s13199-012-0208-9

[B39] Wagg C. Bender S. F. Widmer F. van der Heijden M. G. A. (2014). Soil biodiversity and soil community composition determine ecosystem multifunctionality. Proc. Natl. Acad. Sci. U.S.A. 111, 5266–5270. 10.1073/pnas.1320054111 24639507 PMC3986181

